# Patient acceptability and preferences for communication of coronary artery calcification findings in the NHS targeted lung health checks: a qualitative study

**DOI:** 10.1136/bmjopen-2025-115544

**Published:** 2026-09-08

**Authors:** Abbey Louise Fletcher, Alan Bagnall, Yitka Graham

**Affiliations:** 1 Helen McArdle Nursing and Care Research Institute, University of Sunderland, Sunderland, UK; 2 The Newcastle upon Tyne Hospitals NHS Trust and Newcastle University, Newcastle, UK; 3 Science Complex, University of Sunderland, Department of Pharmacy, Health and Well-being, Sunderland, UK

**Keywords:** QUALITATIVE RESEARCH, Primary Care, Cardiovascular Disease, Patient Preference

## Abstract

**Abstract:**

**Objectives:**

To explore how participants in the UK’s Targeted Lung Health Check (TLHC) programme understand and respond to incidental findings of coronary artery calcification (CAC) and to identify patient preferences for communication that might enhance engagement with cardiovascular prevention such as lipid-lowering therapy (LLT).

**Design:**

Qualitative study using semi-structured interviews and thematic analysis.

**Setting:**

One general practice participating in the National Health Service TLHC programme in North-East England, an area of high social deprivation.

**Participants:**

Ten adults aged 59–71 years with CAC detected on TLHC CT scans and a calculated QRISK >10%.

**Primary outcome:**

To explore patient understanding of incidental CAC findings and how this influences engagement with cardiovascular prevention.

**Secondary outcomes:**

To identify patient preferences for receiving and discussing CAC results, including preferred communication methods, terminology and the role of the clinician in shaping preventive treatment decisions.

**Results:**

Patients initially reported understanding their findings but deeper exploration revealed misconceptions about personal cardiovascular disease risk. A lack of trust in doctors and reliance on non-clinical information sources (social media, family and friends) were barriers to LLT uptake. Trust in the medical professional giving the advice also shaped acceptance of LLT.

**Conclusion:**

Enhancing patient education on CAC findings, fostering doctor-patient trust and tailoring communication strategies may improve LLT uptake. Personalised follow-up and clearer risk explanations could reduce hesitancy and improve engagement with preventive care.

**Strengths and limitations of this study**

- First qualitative study in the UK examining patient perceptions of coronary artery calcification findings within the Targeted Lung Health Check (TLHC) programme.
- Provides detailed insights into how communication format, terminology and clinician trust affect preventive-treatment decisions.
- Includes participants from a socioeconomically deprived region with high cardiovascular disease burden, enhancing policy relevance.
- Small single-site sample limits generalisability but allows for rich thematic depth.
- Findings highlight actionable communication improvements for national TLHC implementation.

## Introduction

 Cardiovascular disease (CVD) is the leading cause of mortality in the UK, responsible for 25% of all deaths.^1^ Socioeconomic deprivation is a major contributor, with higher rates of CVD and poorer health outcomes observed in disadvantaged communities.^2^ The National Health Service (NHS) has identified CVD prevention as a key priority over the next decade, with early risk assessment and intervention playing a crucial role in reducing death from CVD.^3^

The Targeted Lung Health Check (TLHC) was implemented by the NHS to improve early detection, diagnosis, and survival of lung cancers^4^ and was designed to detect lung cancer in high-risk individuals. Within TLHC pathways, low-dose non-gated thoracic CT scans are reviewed by trained radiologists experienced in thoracic imaging. Coronary artery calcification (CAC) is visually assessed and documented within standardised radiology reports. Participants identified as being at elevated cardiovascular risk may subsequently be advised to discuss preventive interventions, including statin therapy, with their general practitioner (GP).

Initial results identified CAC as an incidental finding in over 70% of participants.^5^ CAC is a well-established marker of subclinical atherosclerosis and is strongly correlated with future cardiovascular events.^6^ While the UK National Institute for Health and Care Excellence (NICE) guidelines recommend primary prevention for individuals with a >10% 10-year CVD risk,^7^ uptake of lipid-lowering therapy (LLT) remains suboptimal. Statin hesitancy, the reluctance or refusal of patients to start, continue or properly adhere to prescribed statin therapy for lowering cholesterol and reducing cardiovascular risks, is a persistent challenge, influenced by factors such as misinformation, side effect concerns and distrust of medical professionals.^8 9^

Existing research suggests that CAC visualisation may enhance risk perception and encourage preventive behaviours.^6^ However, little is known about how patients respond to these incidental findings and what communication strategies may optimise engagement with preventive treatments.

This study therefore explores:

1. How patients perceive and understand incidental CAC findings after TLHC.
2. Their preferences for receiving and discussing such results.
3. How communication influences attitudes toward and uptake of LLT.

By focusing on patient perspectives, this work aimed to inform communication practices that support CVD prevention within the TLHC pathway.

## Methods

### Study design

An exploratory qualitative methodology using a thematic analytic framework was chosen for its rigorous six-step method (table 1) and its ability to identify areas of salience to participants to understand their lived experiences. A thematic analysis analytic framework encourages reflexivity and is a widely accepted method for analysing qualitative data, with a structured but flexible approach to identifying, analysing and reporting patterns or themes within a dataset.^10^

**Table 1:** Thematic analytic framework

Step	Action
Familiarisation with the data	Reading and re-reading transcripts and comparing
Generating initial codes	Identifying areas of importance to participants through coding
Generating themes	Grouping codes into themes to identify patterns in the data
Reviewing themes	Checking themes against interview data to ensure themes are representative of participant experiences
Defining and naming themes	Clearly defining and naming each theme
Writing	Writing the data analysis which illuminates the findings

Semi-structured interviews enabled flexible exploration among participants of their understanding of CAC results, emotional responses and attitudes toward preventive treatment (online supplemental material file 2) for the interview topic guide. Data collection and analysis occurred concurrently, allowing emerging themes to inform ongoing analysis. Saturation was assessed iteratively by the research team and was considered achieved when no substantively new themes emerged from subsequent interviews. Following thematic sufficiency identified after approximately eight interviews, two further interviews were conducted to confirm saturation.

We used the Standards for Reporting Qualitative Research (SRQR) reporting guideline^11^to draft this manuscript, and the SRQR reporting checklist^12^when editing, included in online supplemental supplemental A.

### Reflexivity

The research team acknowledged they brought clinical and subject expertise to the study, and from the outset, this was acknowledged in terms of casting assumptions and bias on the data. To reduce the risks of this happening, we embedded reflexive practices during the study. For example, the topic guide was discussed with the research team, but ALF tested this with a representative sample, so the prompts were understood and ensured that participants shaped the process. When analysing the data, the concepts and codes were discussed, and the process was shaped by consensus and discussion of potential biases. We acknowledge that it is impossible not to bring biases to a study, but by reflecting on these, we are confident that this was reduced. This is acknowledged in the limitations of the study.

### Participants

Ten patients aged 59–71 years (6 men, 4 women) were recruited from a single general practice via their GP in North-East England serving a socioeconomically deprived population who were part of the TLHC programme. Eligibility criteria included:

- Participation in the NHS TLHC programme.
- Detection of CAC on CT scan.
- A QRISK >10%.
- No current contraindication to statin therapy.

Each participant had received a TLHC results letter describing CAC findings (online supplemental appendix 1) and advice to contact their GP. All participants received study information, were given time to think about whether they wished to take part, were encouraged to discuss participation with the chief investigator and provided written consent prior to being interviewed.

### Data collection

Interviews were conducted remotely or face-to-face according to preference, lasting 30–60 min. A topic guide was used for the interview which explored the process of obtaining the results of the THLC and subsequent actions as follows:

1. Initial reactions to the CAC result.
2. Understanding of its implications.
3. Sources of additional information.
4. Views on communication methods.
5. Attitudes toward LLT and lifestyle modification.

All interviews were audio-recorded, transcribed verbatim, and anonymised. Data collection took place between October 2023 and March 2024.

### Data analysis

Data were analysed using reflexive thematic analysis (Braun and Clarke).^13^ Two researchers (ALF and YG) independently coded three transcripts, compared the coding and resolved discrepancies through discussion. The remaining transcripts were coded by ALF, with regular debriefing meetings. An audit trail was maintained.

### Ethical considerations

Ethical approval was granted by the Health Research Authority (Reference: 23/NS/0068). All participants provided written informed consent. Identifying information was removed during transcription.

### Patient and public engagement statement

Patients or the public were not involved in the design, conduct, reporting, or dissemination plans of our research.

## Results

The thematic analysis identified four key themes and subthemes, illustrated by in vivo quotes from participant narratives (table 2):

### Theme 1: delivery of findings

**Table 2:** Four key themes and associated subthemes as identified by the thematic analysis

Theme	Subtheme
Exploring delivery of findings	Understanding of CAC findings needing clarity
Identifying preferences for communication	Feeling that the severity of findings mediates preferences; feeling connected to sources of support; ‘seeing’ the problem
Interpreting perceptions of risk	Feeling motivated to engage with treatment
Action and engagement with prevention	Knowledge of preventive treatment; having trust in medical professionals

CAC, coronary artery calcification.

There were mixed responses to the current delivery of CAC findings, and the understanding of the findings in their current format was not consistent.

#### Subtheme: understanding of CAC findings

Patients initially expressed confidence in their understanding but later revealed gaps in knowledge, particularly around medical terminology and implications for their health.

Oh, it was great. It was a great way to inform people… oh, quite easily, the way they were described. – 01

It just described everything, and it was all clear, so it was very understandable. – 03

They were just straightforward, the results, and that was it. – 08

However, further discussion revealed uncertainty:

I understood alright… what is calcification anyway? – 09

I did understand them. I wasn’t 100% sure, but I understood what it was saying. – 05

#### Subtheme: needing clarity

Some participants felt the terminology in their letter was unclear, leading them to seek information online, often increasing anxiety.

…when you’re told there’s, something showing up you just go into free—well not freefall but you’re starting to think, ‘oh my god, is it cancer? – 04

…because when you get a letter and you read something like that which you don’t know, you start googling things, and you know Google, it just gives you the worst. – 05

Patients also expressed frustration over a lack of personalised follow-up:

So, at that time, I thought somebody, a doctor or somebody would sit down and talk to us about it. Nothing was done at all. – 10

…it doesn’t tell you how serious it is or what’s going to happen or what the next steps are or anything. – 05

### Theme 2: preferences for communication

Patients expressed different needs and preferences regarding communication and feel that there is no ‘one-size-fits-all’ approach to communicating clinical findings.

#### Subtheme: feeling that the severity of findings mediates preferences

Most patients preferred letters for routine findings but wanted face-to-face discussions for serious results.

If it was serious, aye, if you needed a doctor to explain if you had problems. – 08

If it was serious… if it had been a growth on my lung or something like that, which looked like cancer, I mean rather than find out about that in a letter, I think it would be better to be told face-to-face by a doctor. – 07

#### Subtheme: feeling connected to sources of support

With respect to how results were communicated, some supported having virtual consultations, but others found them impersonal or impractical.

Yes, I wouldn’t mind that, that would be acceptable, yes, because you’re still seeing the person you’re talking to and you’ve got that connection, you know. – 04

Wanting face-to-face support

It’s just not personal… we’re human beings, not robots. – 10

#### Subtheme: ‘seeing’ the problem

Responses to visual aids were mixed:

I think if you see something visually instead of somebody telling you, you remember it better. – 01

There was reported ambivalence towards visual aids as support:

I don’t think I’d take it seriously. – 02

### Theme 3: perception of risk

The understanding of risk varied between individuals, particularly with respect to how the discovery of CAC impacted on their risk profile and future risk of CVD events.

So obviously, if I don’t look after myself there’s a risk that I could become seriously unwell. – 05

There was a feeling of risk being ever present in all situations, not only with CVD events:

I suppose with anything, there’s a little bit of a risk. – 03

#### Subtheme: feeling motivated to engage with treatment

Patients motivated by family responsibilities appeared to be more proactive:

Not a day goes by that I obviously don’t think about that, because I have got a family and obviously a grandchild and a husband and that, you know? – 05

### Theme 4: action and engagement with prevention

There was variance in understanding and knowledge of interventions which was interpreted in several ways:

#### Subtheme: knowledge of preventive treatment

Patients were aware of lifestyle changes but had limited understanding of statins.

Well, preventative treatment is keeping ourselves healthy and fit. – 08

#### Subtheme: having trust in medical professionals

Trust in doctors was a key factor in statin uptake:

Just the way Dr X explained it… he just told us straight. He just told us exactly what the findings were, why it was recommended, and why he thought it would be suitable for me. And I was quite happy with his explanations. Because I had known him for years, and he’s a doctor that we trust. – 02

However, some were sceptical:

I think it’s a con to tell you the truth… you hear some different reports about them, some people have different reactions to them*.* – 08

## Discussion

### Key findings and interpretation

This study highlights significant gaps in understanding of CAC findings among patients, with many participants overestimating or underestimating their personal CVD risk. While patients initially reported confidence in understanding their results when delivered by letter, further discussion revealed confusion regarding medical terminology and risk severity. This aligns with previous studies demonstrating that complex medical information, particularly in the context of asymptomatic conditions, is frequently misunderstood by patients.^13 14^

A key barrier to LLT uptake was distrust in healthcare professionals, with some patients perceiving statin prescriptions as unnecessary or financially motivated. This scepticism is consistent with previous findings, where patients expressed concerns that statins were overprescribed and questioned their long-term safety.^6 7^ Additionally, misinformation from media and non-clinical sources further reinforced statin hesitancy.^15^

Patients who had previously experienced a cardiovascular event or had a strong sense of familial responsibility were more likely to engage with preventive measures. This supports existing research indicating that personal experiences with illness enhance risk perception and motivate behavioural change.^16^

### Comparison with existing literature

These findings are consistent with previous studies demonstrating challenges in communicating asymptomatic cardiovascular risk and the importance of clinician-patient trust in preventive treatment uptake.^17^ Existing evidence also suggests that misunderstandings regarding statin safety and effectiveness may contribute to reluctance toward LLT, suggesting that improved education on the benefits of LLT could mitigate hesitancy.^18^

Prior research indicates that visual aids and personalised consultations improve understanding and decision-making among patients.^19^ Although some participants in our study believed visual aids might improve understanding, views were mixed. Additional research is therefore needed to evaluate whether visual approaches improve comprehension and engagement within TLHC settings.

### Clinical implications and recommendations

Our findings suggest that clearer explanation of CAC findings and opportunities for personalised discussion may improve patient understanding and informed decision-making regarding cardiovascular prevention.

Future research should evaluate:

- Whether visual aids improve comprehension of CAC findings.
- How communication approaches influence LLT uptake.
- Whether continuity of care influences trust and preventive engagement.

Communication strategies should remain flexible and responsive to differing patient preferences and levels of health literacy.

### Strengths and limitations

This study provides novel insight into patient experiences of incidental CAC findings within the NHS TLHC programme.

However, several limitations should be acknowledged. The sample was small and recruited from a single general practice in a socioeconomically deprived region, which may limit transferability to other settings or populations. Participants were self-selected and may have been more engaged with their health than non-responders. Social desirability bias may have influenced reports of trust in doctors or intention to take statins.

Although thematic saturation was assessed iteratively during analysis, findings should be interpreted as exploratory and context-specific rather than statistically generalisable. Social desirability bias may also have influenced participant reporting regarding trust in healthcare professionals and willingness to engage with treatment.

## Conclusion

Patient understanding of incidental CAC findings identified during TLHC screening is variable and may influence engagement with cardiovascular prevention strategies. Confusion regarding CAC terminology, differing perceptions of cardiovascular risk and mistrust of preventive medication may contribute to statin hesitancy.

Improved communication approaches and opportunities for personalised discussion may support informed decision-making regarding cardiovascular prevention. Further multi-site research is needed to evaluate interventions designed to optimise CAC communication within TLHC pathways.

## Supplementary material

10.1136/bmjopen-2025-115544 online supplemental file 1

10.1136/bmjopen-2025-115544 online supplemental file 2

10.1136/bmjopen-2025-115544 online supplemental file 3

## Data Availability

Data are available upon reasonable request.
