# Supplementary material for: Patient acceptability and preferences for communication of coronary artery calcification findings in the NHS targeted lung health checks: a qualitative study
Source: BMJ Open. 2026 Sep 8;16(9):e115544. doi: 10.1136/bmjopen-2025-115544 (PMC13561021; doi:10.1136/bmjopen-2025-115544)
Supplement: Supplementary file 1 [file bmjopen-2025-115544.reviewer_comments.pdf]

PEER REVIEW HISTORY

BMJ Open publishes all reviews undertaken for accepted manuscripts. Reviewers are asked to complete a checklist review form and are provided with free text boxes to elaborate on their assessment. These free text comments are reproduced below.

ARTICLE DETAILS

Title (Provisional)

Patient Acceptability and Preferences for Communication of Coronary Artery Calcification Findings in Targeted Lung Health Checks: A Qualitative Study

Authors

Fletcher, Abbey Louise; Bagnall, Alan; Graham, Yitka

VERSION 1 - REVIEW

**Reviewer**	**1**
**Name**	**Yasa, Ketut Putu**
**Affiliation**	**Udayana University Faculty of Medicine, Cardiothoracic and Vascular Surgery Division, Department of Surgery/Prof. Dr. I.G.N.G. General Hospital, Denpasar, Bali Indonesia**
**Date**	**08-Mar-2026**
**COI**	**None**

1. Regarding CAC result, please explain how does it process , how does it performed, who is expertise the CT scan result, what kind of Ct scan . It could add as short explanation about TLHC Program in introduction

2. Explain statin treatment policy of participant

3. How is Sample size method, ten participant is good enough ?

**Reviewer**	**2**
**Name**	**El-Mughayyar, Dana**
**Affiliation**	**New Brunswick Heart Centre, Cardiac Surgery**
**Date**	**16-Apr-2026**
**COI**	**None**

Thank you for your submission and congratulations on your work.

This a well-conducted qualitative study addressing an important and underexplored topic on patient understanding and communication of CAC findings in TLHC. Your research question is clear; study design (semi-structured interviews + thematic analysis) is appropriate. Results are clearly presented with coherent themes supported by participant quotes; conclusions are justified.

Please consider the following for your manuscript:

-Methods could include slightly more detail on the interview guide and coding process to improve reproducibility.

-Limitations section could be expanded to better address potential biases (e.g., recruitment via GP, sample homogeneity) and transferability.

- The reference section style varies (ref #4 has citing date and the others don't)? Please follow BMJ Open guidelines.

**Reviewer**	**3**
**Name**	**Rahouma, Mohamed**
**Affiliation**	**Weill Cornell Medicine, Cardiothoracic Surgery**
**Date**	**17-Apr-2026**
**COI**	**None**

The authors reported their work entitled "Patient Acceptability and Preferences for Communication of Coronary Artery Calcification Findings in Targeted Lung Health Checks: A Qualitative Study". This is a well-written, timely, and relevant qualitative study exploring patient perspectives on incidental coronary artery calcification (CAC) findings identified through the NHS Targeted Lung Health Check (TLHC) programme. The study addresses an important gap in understanding how communication influences engagement with cardiovascular prevention, particularly statin therapy. The findings are insightful and have clear implications for clinical practice and policy. I have the following comments:

Major Comments

1. Sample and Generalisability: The sample is small (n=10), drawn from a single general practice in a socio-economically deprived region. While the authors acknowledge this as a limitation, the claim of “data saturation” is not fully justified. Please provide a more detailed rationale for saturation (e.g., how it was assessed, after how many interviews, by whom). Consider toning down claims of transferability unless further multi-site data are presented.

2. Methods – Missing Details: The topic guide is mentioned but not included in the main text or appendices (only referenced as “Appendix 2” which is not present in the proof). Please include the full interview topic guide as a supplementary file or appendix. This is essential for transparency and replicability.

3. Reflexivity: While the SRQR checklist mentions researcher characteristics, the main text lacks a dedicated reflexivity statement. The influence of clinicians (e.g., AB being a consultant) on data interpretation is not critically examined. Please add a short reflexivity subsection in Methods, discussing how researchers' backgrounds, assumptions, and relationships with participants may have shaped data collection and analysis.

4. Analysis – Dual Coding and Consensus: The authors state that "both researchers were involved in coding" but do not specify whether coding was independent, how disagreements were resolved, or whether inter-coder reliability was assessed (even qualitatively). Please clarify the coding process, including whether a coding framework was developed iteratively, how many researchers coded each transcript, and how consensus was achieved.

5. Results – Overclaiming and Discrepancies: Some quotes appear contradictory. For example, DA01 and SH03 express high satisfaction with letter clarity, while KH09 and AF05 express confusion. The analysis does not fully explore or explain this divergence. Please acknowledge and interpret this inconsistency more explicitly. Consider whether it reflects different health literacy levels, cognitive biases, or social desirability bias.

6. Discussion – Recommendations Outweigh Evidence: The authors make strong recommendations (e.g., "visual aids may be beneficial," "greater continuity of care may foster trust") that, while reasonable, go beyond what the data directly support. No intervention was tested. Please reframe these as hypotheses or implications for future research and service design, not as evidence-based recommendations.

Minor Comments

7. Duplicate Content: Thematic analysis table (Braun & Clarke steps) and themes table appear twice (pages 25 and 31). The SRQR checklist also appears twice (pages 26–30 and 33–36).

Recommendation: Remove duplicate tables and check for other redundancies. This suggests a compilation error during proofing.

8. Writing and Clarity

Typo: Page 11 – "AF05" appears twice in a quote block.

Awkward phrasing: Page 13 – "There was reported ambivalence" → simplify to "Some participants were ambivalent."

Jargon: Page 15 – "statin hesitancy" is used repeatedly; consider defining it early.

9. References

Issue: Reference 19 (Haslam, 2014) appears incomplete

Recommendation: Verify and update all references, especially those cited as key evidence (e.g., statin hesitancy, risk perception).

10. Ethical Approval

Issue: Ethical approval reference is given, but no mention of how participants were reimbursed (if at all) or whether distress protocols were in place.

Recommendation: Add a brief note on participant reimbursement and emotional safeguarding, as is standard for qualitative health research.

Suggested Revisions for Key Sections

Abstract – Primary Outcome: The current wording (“To explore how participants… understand…”) is not a primary outcome but an objective. Rephrase to: To explore patient understanding of incidental CAC findings and how this influences engagement with cardiovascular prevention.

Methods – Data Analysis: Add:

Data were analyzed using reflexive thematic analysis (Braun & Clarke, 2006). Two researchers (AF and YG) independently coded three transcripts, compared coding, and resolved discrepancies through discussion. The remaining transcripts were coded by AF, with regular debriefing meetings. An audit trail was maintained.

Discussion – Limitations: Add:

Participants were self-selected and may have been more engaged with their health than non-responders. Social desirability bias may have influenced reports of trust in doctors or intention to take statins.

VERSION 1 - AUTHOR RESPONSE

REVIEWER 1

Comment 1: Regarding CAC result, please explain how it is processed, how it is performed, who reports the CT scan result, and what type of CT scan was used. It could also be added as a short explanation about the TLHC Programme in the introduction.

Response:
Thank you for this helpful suggestion. We agree that additional context regarding the Targeted Lung Health Check (TLHC) programme and coronary artery calcification (CAC) assessment would improve clarity. We have expanded the Introduction to include a brief description of the TLHC programme. (Line 100)

Comment 2: Explain statin treatment policy of participants.

Response:
Thank you for this comment. We have expanded the Introduction section to clarify the clinical pathway following identification of CAC findings, including current guidance regarding statin initiation. (Line 102)

Comment 3: How was the sample size determined? Is ten participants sufficient?

Response:
Thank you for raising this important point. We have expanded the Methods section to explain our sampling approach and provide further detail regarding how data saturation was assessed. We have also clarified that recruitment ceased when no substantially new themes were emerging from subsequent interviews. Additional discussion regarding the strengths and limitations of the sample size has been added to the Discussion section. (Line 210).

REVIEWER 2

Comment 1: Methods could include slightly more detail on the interview guide and coding process to improve reproducibility.

Response:
We thank the reviewer for this suggestion. We have expanded the Methods section to provide additional detail regarding the development and content of the semi-structured interview guide. We have also included a more detailed description of the coding process. Furthermore, the full interview guide has been provided as a supplementary appendix.

Comment 2: Limitations section could be expanded to better address potential biases and transferability.

Response:
Thank you for this important observation. We have expanded the Limitations section to discuss potential recruitment bias associated with general practice-based recruitment, the relatively homogeneous sample, possible self-selection bias, and factors affecting transferability of the findings. We have also emphasised that the findings should be interpreted within the context of the study setting. (Line 440)

Comment 3: Reference style varies.

Response:
Thank you for identifying this inconsistency. We have reviewed and revised all references to ensure they conform fully with BMJ Open formatting requirements.

REVIEWER 3

Major Comment 1: Sample and Generalisability.

Response:
We appreciate this important comment. We agree that qualitative studies require careful consideration of saturation and transferability. We have expanded the Methods section to provide a detailed description of how thematic saturation was assessed, including the point at which no substantially new themes were identified. We have also revised the Discussion to moderate claims regarding transferability and emphasise that findings are most applicable to similar TLHC populations and healthcare settings.

Major Comment 2: Interview topic guide missing.

Response:
Thank you for identifying this omission. The full interview topic guide has now been included as a supplementary appendix and is referenced appropriately within the manuscript.

Major Comment 3: Reflexivity.

Response:
We agree that greater transparency regarding reflexivity strengthens qualitative research reporting. We have added a dedicated reflexivity subsection within the Methods section outlining the professional backgrounds of the research team, potential assumptions and perspectives relevant to the study topic, and the steps taken to minimise undue influence on data collection and interpretation. (Line 162)

Major Comment 4: Dual coding and consensus.

Response:
Thank you for this suggestion. We have expanded the data analysis section to clarify the coding process. Specifically, we now describe how transcripts were coded, whether coding was conducted independently, how discrepancies were discussed and resolved, and how themes were iteratively refined through regular meetings among the research team. We have also clarified the role of each researcher throughout the analytic process. (Line 210)

Major Comment 5: Results – Divergent participant views.

Response:
We appreciate this thoughtful observation. We agree that the variation in participants' responses is important and informative. We have revised the Results section to explicitly acknowledge differences in participants' understanding and perceptions of the communication materials. We have also expanded the Discussion to consider potential explanations for these differences, including variation in health literacy, prior knowledge, personal experiences, and interpretation of risk information. We have taken care not to overstate conclusions beyond the data. (Line 232)

Major Comment 6: Discussion – Recommendations outweigh evidence.

Response:
Thank you for this valuable comment. We agree that some statements could be interpreted as recommendations beyond the direct evidence generated by this study. We have revised the Discussion to frame these points more cautiously as potential implications for future service development and hypotheses for future research rather than evidence-based recommendations. The wording throughout has been adjusted accordingly. (Line 407)

Minor Comment 7: Duplicate content.

Response:
Thank you for identifying these duplication errors. These arose during manuscript compilation. The duplicate tables and SRQR checklist have been removed and the manuscript has been carefully checked for any further redundancies.

Minor Comment 8: Writing and clarity.

Response:
Thank you for these observations. The duplicate participant identifier has been corrected, the wording regarding ambivalence has been simplified, and the term "statin hesitancy" is now defined upon first use. (Line 110)

Minor Comment 9: References.

Response:
Thank you for identifying this issue. We have reviewed all references and corrected the incomplete citation. We have also undertaken a full reference check to ensure accuracy and completeness.

Minor Comment 10: Ethical approval.

Response:
We thank the reviewer for this suggestion. We have expanded the Ethics section to clarify whether participants received reimbursement and to describe procedures used to support participant wellbeing during interviews, including the management of any distress arising during discussion of health-related experiences.

Suggested Revision – Abstract.

Response:
Thank you for this suggestion. We have revised the abstract wording to better reflect the study objective and focus on patient understanding of CAC findings and engagement with cardiovascular prevention. (Line 40)

Suggested Revision – Data Analysis.

Response:
We appreciate this recommendation. The Data Analysis section has been revised to provide a more detailed account of coding procedures, researcher involvement, consensus processes, and maintenance of an audit trail. (Line 212)

Suggested Revision – Limitations.

Response:
Thank you for this helpful suggestion. We have incorporated additional discussion regarding self-selection bias, participant engagement, and the potential influence of social desirability bias on reported attitudes towards clinicians and statin therapy. (Line 444)

VERSION 2 - REVIEW

**Reviewer**	**3**
**Name**	**Rahouma, Mohamed**
**Affiliation**	**Weill Cornell Medicine, Cardiothoracic Surgery**
**Date**	**27-Jun-2026**

COI

The authors addressed the prior comments properly.
