# Supplementary material for: Patient acceptability and preferences for communication of coronary artery calcification findings in the NHS targeted lung health checks: a qualitative study
Source: BMJ Open. 2026 Sep 8;16(9):e115544. doi: 10.1136/bmjopen-2025-115544 (PMC13561021; doi:10.1136/bmjopen-2025-115544)
Supplement: Supplementary file 2 [file bmjopen-16-9-s001.docx]

**Appendix 1: Example TLHC Patient Letter**

**CORONARY ARTERY CALCIFICATION**

Your scan showed that you have some calcification of the blood vessels supplying your heart. Calcification is one of the earliest signs of fatty deposits (atherosclerosis) in heart vessels. When we see calcium, it tells us that you are likely to benefit from taking cholesterol-lowering medication and making lifestyle changes to reduce your risk of having heart attacks or stroke within the next 10 years.

Lifestyle changes include not smoking, being careful not to be overweight, being physically active, and ensuring other medical problems such as high cholesterol, diabetes, and high blood pressure are well controlled. Your GP will be happy to work with you to check for or help control these medical problems.

If you are already taking a statin, or have been previously diagnosed with angina, heart attack, stroke, or ischemic heart disease, then you do not need to do anything else.

If you are worried about symptoms of chest pain or chest tightness and breathlessness when exerting yourself, then you should contact your GP or 111 to discuss your symptoms.

We have written to your GP to explain the scan findings. If you are not already taking a statin, you will be contacted by your surgery to discuss how you may be able to reduce your risk of future heart disease or stroke.
