# Supplementary material for: Patient acceptability and preferences for communication of coronary artery calcification findings in the NHS targeted lung health checks: a qualitative study
Source: BMJ Open. 2026 Sep 8;16(9):e115544. doi: 10.1136/bmjopen-2025-115544 (PMC13561021; doi:10.1136/bmjopen-2025-115544)
Supplement: Supplementary file 4 [file bmjopen-16-9-s003.docx]

14^th^ June 2023

Version 1

IRAS ID: 319742

**Title:** Incidental Coronary Artery Calcium during Targeted Lung Health Checks (TLHC): Understanding Patient Perspectives to Determine Acceptability and Preferences For Presentation of Incidental Findings of Coronary Artery Disease

**Chief Investigator: Abbey Fletcher**

**Interview Topic Guide - Prompts**

Personal experience of being presented with TLHC findings and associated risk

- Can you tell me your own experience of being presented with the findings of the TLHC scans?
- How well did you understand the findings presented?

Understanding of risk

- What does the term “risk” mean to you?
- Do you feel you understood your personal risk identified by the TLHC?
- Why do you feel you did/did not understand?
- Do you feel you are at risk? Why?

Preferences for delivery of findings

- Can you think of any way to improve the delivery of TLHC findings and associated risk based on your own experience?
- Would you prefer any of the following methods of delivery?
  - Face-to-face
  - Virtually
  - Animation
  - (show participants examples of each method)
- Why?

Preventative Treatment

- What is your experience/knowledge of preventative treatments?
- Would you benefit from more in depth explanation of preventative treatments available to you?
- What would influence your decision around whether to commence preventative treatment?
- Why?

Closing Comments

- From your personal experience, is there anything else I should know so that I can understand you experiences of receiving the findings of the TLHC and associated risk?
- Is there anything else you would like to discuss before we end the interview?
